# What are the characteristics of the health and care workforce supporting people living with frailty in England now and what is needed for the future? A national survey

**DOI:** 10.1136/bmjopen-2026-116867

**Published:** 2026-07-01

**Authors:** Carole Fogg, Tracey England, Martin Vernon, Jane Ball, Lee-Ann Fenge, Peter Griffiths, Francesca Lambert, Abigail Barkham, Sally C Brailsford, Vivienne Windle, Bronagh Walsh

**Affiliations:** 1 School of Health Sciences, University of Southampton, Southampton, UK; 2 Tameside and Glossop Integrated Care NHS Foundation Trust, Ashton-under-Lyne, UK; 3 Royal College of Nursing Institute of Nursing Excellence, London, UK; 4 Faculty of Health, Environment and Medical Sciences, Bournemouth University, Bournemouth, UK; 5 National Institute for Health Research Applied Research Collaboration, Wessex, Hampshire, UK; 6 Hampshire and Isle of Wight Healthcare NHS Foundation Trust, Southampton, UK; 7 Business School, University of Southampton, Southampton, UK; 8 Public Contributor, Southampton, UK

**Keywords:** Frailty, Health Services, Surveys and Questionnaires, GERIATRIC MEDICINE, Health policy

## Abstract

**Abstract:**

**Background:**

Frailty is a common condition in older adults which becomes more prevalent and more severe with age. Health and care services designed to meet the needs of older adults living with frailty are expanding in number and scope, but information on the workforce needed to deliver services both now and in the future is lacking.

**Objectives:**

To understand the service design and staffing configurations for frailty services through gathering data on the setting and purpose of services; target population; referral methods into the service; specific activities delivered; frailty assessment tools; key service and patient outcomes; staff involved; future service priorities and anticipated future workforce requirements.

**Design:**

National survey, circulated electronically via national networks and organisations involved in the care of patients with frailty (n=26).

**Setting:**

England health and care settings.

**Participants:**

Health and care professionals delivering services for people living with frailty.

**Results:**

There were 93 survey responses from frailty services across England, of which 82 contained usable information. Respondents included clinicians and managers in a range of health and care sectors and the voluntary sector. Frailty services across settings commonly prioritised reducing frailty-associated risks but few focused on prevention. Staff teams included representation across professions, with specialists in older people’s care (eg, geriatricians, advanced practitioners) present in most teams, but non-specialist team members (eg, therapists, social workers and care co-ordinators) comprised a large proportion of the total workforce. All respondents identified similar priorities for their service in future, including reducing frailty progression, and specified needs for additional staff which broadly reflected the current team configurations. However, staff vacancies or unmet patient need due to low capacity was highlighted, and all respondents identified the need for additional staff in future.

**Conclusions:**

Services designed to identify and manage people with frailty are complex and require a workforce with specialist training to assess, plan and deliver care. Current services are understaffed with insufficient capability to prevent frailty onset or slow progression, thereby failing to address unmet need. Workforce planning and resourcing to address frailty-related needs is urgently required.

**Strengths and limitations of this study**

- The survey gathered detailed information about the current and future priorities, activities of frailty services and varied workforce configurations across different settings.
- To maintain anonymity of the respondent, specific service names or National Health Service (NHS) Trusts were not requested; however, region and care setting were used to characterise diversity of type and location of services.
- To maintain the focus on the service rather than individuals and ensure responses were not identifiable, data on the range of professionals and expertise involved were gathered rather than description of specific staff complements.

## Introduction

 Frailty is a complex condition characterised by increasing vulnerability to health stressors, which becomes more common and increases in severity with age. Its prevalence in ageing populations globally will continue to be a major driver of increased health and care service use and costs over the coming decades.^1^,^3^ Services in the UK are already finding it hard to meet current demand,^4^ so there is an urgent need to understand how future demand associated with frailty can be managed successfully. Although health and care services designed to meet the needs of people living with frailty are expanding in number and scope to meet these growing demands,^5^ our scoping review found that information on the workforce needed to deliver these services is lacking.^6^ This makes it challenging to define a ‘gold standard’ in frailty services, where workforce capacity and capability is a key consideration.

Remodelling and integration of health and care systems to meet the predicted future needs of older people living with frailty is challenging.^7 8^ However, frailty service development is vital to manage predicted demand for urgent, emergency and social care services.^9^ This will require care options to maintain independence at home and prevent illness, including involvement of social care in frailty prevention, systematised use of Comprehensive Geriatric Assessments, increasing use of technology (eg, wearables and monitors), with implications for the associated workforce required to support these developments.^10 11^ Older adults express a preference for ‘Ageing in Place’^12^,^14^; however, this requires provision of sustainable health and care services to enable them to stay safely at home and receive appropriate care in their communities. The nature of care needs differs due to personal complexities, social and familial situations^15 16^ which evolve with frailty progression. Global estimates suggest two thirds of people who reach older age are likely to require long-term support and care from others to perform basic activities of daily living, but only one in four countries have enough financial and political resources to support care integration responsive to their needs.^17^

As guidance on clinical care for people living with frailty has been implemented in the UK,^18^ frailty services have expanded in diverse ways, but there is a lack of information on what services are being provided or planned and how they are staffed.^6^ Given the finite pool of health and social care staff, and the impact of ageing populations on workforce availability, changes to service configurations are likely to involve trade-offs of staff between services in different sectors. Understanding which staff are needed to deliver different frailty services is essential. Management of frailty requires the involvement of staff with specialist skills, including geriatricians and advanced clinical practitioners (ACPs), as well as involvement of a range of professions and cadres of staff with generic skills in older people’s care. Projections of future need signify large recruitment and training challenges^19 20^; it is estimated that globally less than 60% of countries include long-term care in their national competency framework for geriatric care workers.^17^ However, these projections are based on the continuation of present service and workforce configurations, whereas planned shifts in care focus for older people towards communities must inform projections based on new and planned services.

Our wider project (Planning for Frailty: Optimal Health and Social Care Workforce Organisation Using Demand-led Simulation Modelling (FLOWS))^21^ will use information from this survey as well as a variety of other sources to simulate future service demand and workforce needs to deliver care for people living with frailty. The purpose of this survey was to add to our current understanding from the literature about frailty workforce, to explore the range of frailty-specific services that are currently provided or planned in health and social care organisations, and describe staffing configurations in terms of numbers, competencies and skills. The survey also provided an opportunity to explore how these services could be staffed in future and to identify potential gaps in workforce provision.

## Methods

### Aim

To describe the workforce configuration and activities of frailty-specific services within a range of services for older people in different health and social care settings, patient caseloads and future priorities for these services with predicted workforce needs.

### Design

Cross-sectional survey, distributed electronically.

### Setting

Health and care services in England, UK.

### Survey development

The survey was developed through a consultative process with the full research team and the FLOWS project stakeholder group,^21^ using information gathered from a scoping review^6^ and previous workforce surveys.^22 23^ The survey was piloted by clinical members of the team working in different roles (geriatrician, advanced nurse practitioner) in different environments (acute and community trusts) and modified according to feedback.

### Participants

Participant eligibility was ascertained by a question following the information sheet explaining the purpose and nature of the study and prior to asking for consent to participate, that is, “Are you involved in the provision of a service for older people that are living with frailty?”. It was necessary to keep this question intentionally broad as services for people with frailty are diverse and have developed in different ways according to local needs and other features of local health and care systems. This question therefore allowed the respondent to decide whether or not their services were applicable. We aimed for a sample size of 200, based on previous national surveys relating to workforce and adjusted for the different target population and recruitment method.^22 24^

### Survey description

The survey was divided into three main sections. The first section asked about the respondent’s role in the service, including details on their professional group, years of experience of working with older people with frailty, time commitment in the service, how long the service has been in operation, National Health Service (NHS) region, provider type (eg, acute or community setting) and specialist qualifications. To maintain confidentiality, closed-category survey questions relating to the individual were used, rather than details on the specific roles, employers or other potentially identifiable information. The second section collected details about the service itself, including the patient group, the main activities, tools used to identify frailty, details on the patient pathway into the service, average patient caseloads, key measures of success for patients or the service, and key service priorities over the next 5–10 years. Section three focused on the workforce involved in delivering the service, including the different health and care professions, whether the staff were sufficient for the level of patient need, details on staff vacancies, and which additional staff resources would be needed to deliver the service 5–10 years into the future. The full survey with the option responses for each question is in online supplemental file 1.

Qualtrics XM software (Utah, USA) was used to create an electronic version of the survey for distribution.

### Distribution

As there is no central register of frailty services in England, professional networks were engaged as an alternative method of distribution and to capture information on services in a wide range of settings. An introductory e-mail and a survey link for circulation was sent to organisations representing health and care professionals who work with older people, for example, Royal Colleges (including those representing pharmacists and allied health professionals (AHPs) including therapists), the British Geriatrics Society, Social Care directors/British Association of Social Workers, and all Applied Research Collaborations which encompass large groupings of staff working in Health and Social Care. There were several rounds of circulation, which also included via networks suggested by our full research team members and those of the Steering committee. A QR (Quick Response) code was also generated for distribution via social media (BlueSky). The survey was open from 1 October 2024 to 30 June 2025.

### Analysis

Data were downloaded from Qualtrics and analysed using Microsoft Excel. As the survey link was sent to unknown participants via organisations, we were unable to prevent multiple participation of participants, and so responses were assessed for potential duplication of participant characteristics (eg, region, profession, role). Although not detectable, it is possible that there may have been more than one respondent from a particular service, which would add to the depth of data from different team member perspectives. Data analysis comprised descriptive statistics (frequencies and percentages, with denominators of either the number of survey respondents or the total number of options ticked as appropriate) and categorisation or narrative description of free-text responses. Smaller frequencies were grouped into ‘other’ where appropriate. Descriptive statistics were used to summarise the range of services and service configurations, including location and setting, type, caseload, staffing and provider types. Summary data on the caseload range by professional staff groups and service setting were calculated. The majority of the analyses were unstratified, as the intention was to explore the diversity of component activities and the related workforce; however, the workforce was presented by care setting (secondary and community care) to understand how specialist and non-specialist staff may be distributed by care sector.

### Patient and public involvement

This survey is part of a larger project which was co-developed with members of the public, patients and carers of people living with frailty. The lead Public Patient Involvement (PPI) representative had input to the survey design and co-authored this paper and will be involved in summarising the full study results in plain English for public dissemination.

## Results

### Description of responses

There were 93 surveys submitted in total, 85 via links sent by e-mail and organisational circulation and 8 via social media. 11 did not meet the eligibility criteria, leaving 82 surveys, of which 72.0% (n=59) were 100% complete; all questions with responses were included in the analysis.

### Respondent professions and qualifications

The majority of respondents were a team member or team leader (table 1); other respondents included directors or clinical leads, assessors within social services, voluntary group organisers and engagement leads. Most team leaders were consultant geriatricians, with advanced clinical practitioners (typically educated to Master’s level) and consultant practitioners (educated to PhD (Doctor of Philosophy) level) also represented. Most respondents (95.3%) had four or more years of experience working with older people living with frailty, and 24.5% had additional training in care of older people or frailty. The range of highest qualifications achieved reflected the spread of roles described, and included General Certificate of Secondary Education (GCSEs), National Vocational Qualifications (NVQ) as well as undergraduate degrees and diplomas, through to postgraduate degrees and diplomas (MSc (Master of Science) or PhD). A variety of specialist training for older people was described, including specialty certificate in geriatric medicine, modules or professional training on frailty or dementia care, gerontology and palliative care. These were grouped into formal qualifications or additional/general/vocational training.

**Table 1:** Survey respondent characteristics

Questions, n responses	Responses	N (%)*
Role within the service (n=75)	Team member	28 (37.3%)
	Team leader	23 (30.7%)
	Individual service provider	6 (8.0%)
	Service manager or director	6 (8.0%)
	Other	12 (16%)
Professional group or background (n=74)	Consultant geriatrician	24 (32.4%)
	Non-specialist nurse or AHP	16 (21.6%)
	Consultant practitioner/specialist nurse or AHP	10 (13.5%)
	Other doctor (eg, GP (primary care doctor), registrar, palliative medicine)	10 (13.5%)
	Social work/social care	8 (10.8%)
	Other	6 (8.1%)
Years of experience working with older people living with frailty (n=64)	More than 10 years	44 (68.8%)
	4–10 years	17 (26.6%)
	Up to 3 years	3 (4.7%)
Specialist qualifications (n=49, 62 options given†)	Formal qualifications (eg, geriatrician, advanced practitioner or nurse consultant)	30 (61.2%)
	General training	13 (26.5%)
	Additional training in older people’s care	12 (24.5%)
	Vocational training (including experience gained ‘on-the-job’)	7 (14.3%)

* Of responses given.

† Multiple qualifications specified by some respondents.

AHP, allied health professional; GP, general practitioner.

Respondents had a range of professional backgrounds, with specialist doctors and nurses and representatives from the major AHP groups included (eg, physiotherapists, speech and language therapists, occupational therapists, pharmacists), in addition to people with social work or social care backgrounds and commissioners and programme co-ordinators.

### Frailty service settings and current priorities

Services in all of the NHS regions in England were represented (table 2). The settings most likely to host frailty services were acute inpatient settings (43.1%) or community care (44.8%), with involvement of primary care, outpatients, community hospitals and the wider care system. Where a service crossed multiple settings, the most common arrangement was between secondary and community care (n=9), with primary and community care being less common (n=2). The majority of services were well established, with three quarters running for more than 5 years. Only a third of services were primarily targeted at people living with frailty and less than 5% for people at risk of developing frailty. However, the majority used a recognised frailty assessment or identification tool. Patients were referred to the service from a broad range of other health and social care sectors (table 2).

**Table 2:** Characteristics of the frailty service

Questions, n responses	Responses	N (%)*
NHS region (n=55)	South East	18 (32.7%)
	North West	13 (23.6%)
	North & East Yorkshire	7 (12.7%)
	London	7 (12.7%)
	South West	6 (10.9%)
	Midlands	3 (5.5%)
	East of England	1 (1.8%)
Settings (n=85, 97 options selected)†	Community care	26 (44.8%)
	Acute hospital (inpatient)	25 (43.1%)
	Primary care	7 (12.1%)
	Acute hospital (outpatient)	6 (10.3%)
	Other (includes hospice, leisure industry)	6 (10.3%)
	Community hospital (inpatient)	5 (8.6%)
	Emergency department	5 (8.6%)
	Community hospital (outpatient)	4 (6.9%)
	Integrated care system	4 (6.9%)
	Charity or voluntary	4 (6.9%)
	Residential or nursing care	3 (5.2%)
	Learning disability services	2 (3.4%)
How long has service been running? (n=64)	More than 5 years	48 (75.0%)
	3–5 years	8 (12.5%)
	1–2 years	7 (10.9%)
	Less than 1 year	1 (1.6%)
Which types of patients is the service for? (n=52)	Older people generally, some of whom could be living with frailty	26 (50.0%)
	Only people identified as living with frailty	14 (26.9%)
	People identified as frail within another service	3 (5.8%)
	Older people at risk of developing frailty	2 (3.8%)
	Other (includes patients with multimorbidity and polypharmacy, palliative care)	7 (13.5%)
Referral source (n=51, 164 options selected)†	General practice (primary care)	38 (74.5%)
	Community services including nursing, hospital-at-home	37 (72.5%)
	Secondary care	28 (54.9%)
	Urgent care (including ambulance services)	25 (49.0%)
	Self-referral	14 (27.5%)
	Social work/social care	14 (27.5%)
	Voluntary services	7 (13.7%)
	Other	1 (2.0%)
Assessing frailty (n=48, 74 options selected)†	Rockwood CFS	40 (83.3%)
	Timed Up and Go Test	13 (27.1%)
	Other	11 (22.9%)
	Electronic Frailty Index (eFI)	7 (14.6%)
	Gait speed	3 (6.3%)
	Fried	0 (0.0%)
	PRISMA-7	0 (0.0%)

* Of responses given.

† Multiple options permitted - % are of total responses.

CFS, Clinical Frailty Scale; NHS, National Health Service; PRISMA-7, Program of Research to Integrate Services for the Maintenance of Autonomy-7.

**Figure 1:**
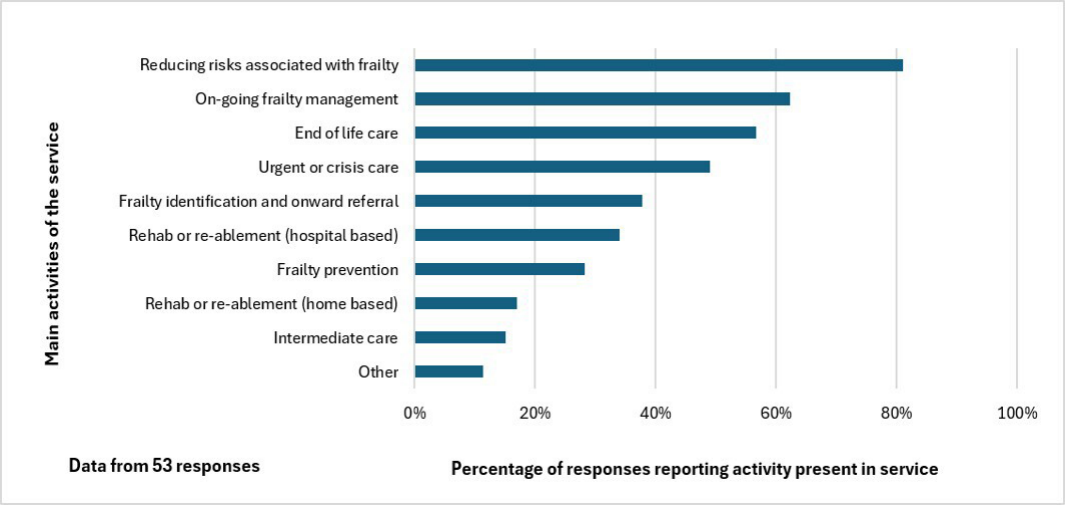
Distribution of main activities of the services.

The most common activity reported was to reduce risks associated with frailty (eg, falls or avoidable hospital admission), followed by on-going frailty management, including specific aspects such as medications management and disease management and assessment (figure 1). End-of-life care (likely to include facilitating discharges to appropriate settings) was also a feature of more than 50% of services, while frailty prevention was less common but was reported in care settings including primary/community care, residential homes and acute hospitals. Additional specific activities included needs assessments and exercise classes.

There was much commonality between the main measures of success reported (n=48). More than 60% of services considered reduced hospital admissions from home, improved patient and/or carer satisfaction, falls risk reduction, medical optimisation, medications management, improved clinical outcomes and maintaining independence at home to be key measures of success. The least common measures of success were reduced risk of new care home admission (35.4%) and reduced frailty progression (29.2%), of which the latter services all had frailty prevention and/or rehabilitation or reablement specified as key activities.

### Current workforce configurations and patient caseloads

Around a third of specialist and non-specialist staff appear to be splitting their time between the frailty service and delivery of other work, by working within the frailty service up to 3 days per week (table 3). Although social services respondents were fewer, they were more likely to be engaged fully within the service.

**Table 3:** Working hours in the frailty service by professional group

Hours per week	N (%)
Geriatrician, specialist nurse or AHP	
Up to 1 day	6 (21.4%)
2–3 days	4 (14.3%)
4–5 days	16 (57.1%)
>5 days	2 (7.1%)
Other doctor/nurse/AHP	
Up to 1 day	3 (13.6%)
2–3 days	5 (22.7%)
4–5 days	14 (63.6%)
Social work /care staff	
Up to 1 day	0
2–3 days	0
4–5 days	3 (100%)
Other staff member	
Up to 1 day	0
2–3 days	3 (37.5%)
4–5 days	5 (62.5%)

Note: Overall total of 61 responses given, n (%) of professional group totals.

AHP, allied health professional.

Reported patient caseloads varied according to the setting (table 4). Acute care had the largest caseloads, with between 50 and 150 patients seen by specialist staff per week in acute frailty services, same-day emergency care (SDEC) or emergency departments, in addition to 38–250 patients on acute wards. Services in community settings varied according to the service context, with 25 patients per week in a community hospital, 50–80 per week in wider community care settings and 40–240 per week in primary care. Doctors, nurses or AHP staff delivering virtual wards had caseloads of around 60 patients per week per team member, and 5–30 patients per week for other community-based AHP staff, pharmacists and palliative care specialists. Primary care estimated three home visits and 10 telephone consultations per week. Other community services had significant caseloads for reablement, care assessments, exercise services and community support workers. Services spanning settings, for example, community/acute and residential care, or based within an integrated care system, had caseloads of 25 per week for specialist geriatric staff, between 2–10 per week for social work/care staff and 10–15 per week for staff within a rehabilitation service.

**Table 4:** Patient caseload estimates by setting

	Acute setting	Community setting	Other
Geriatrician, specialist nurse or AHP	Acute frailty service (includes SDEC): 50–150/week Hospital ED: 20–24/day (100–120 per week) Inpatient wards: 38–250 present daily	Community hospital: 5/day Primary care: 8–30/day ACP team: 10/week per team member Community care: 50–80/week	Service spans community/acute/residential care: 25/week
Other doctor/nurse/AHP	Outpatient clinic: 2–3/week	Virtual ward: 12/day per team member. Specialty-specific services (eg, palliative care, physiotherapy, pharmacy, OT): 5–30/week per staff member. Community out-of-hours (OOH) urgent care response service (UCR): 1/3 of patients Nearly all patients receiving home visits. Primary care: 3 home visits and 10 telephone consultations/week.	
Social work/care staff			Integrated care system: 7–10/week Service spans community care/nursing home: 2–6/week
Other staff member		Reablement service: 74/week Day centre: 60 service users supported by 10 staff Care assessments: 20–26 ongoing Community support worker: 10 or more referrals/week Exercise service: 45/week	Rehabilitation service spans acute hospital and community care: 10–15/week

ACP, advanced clinical practitioner; AHP, allied health professional; ED, Emergency Department; OT, occupational therapy; SDEC, same-day emergency care.

**Figure 2:**
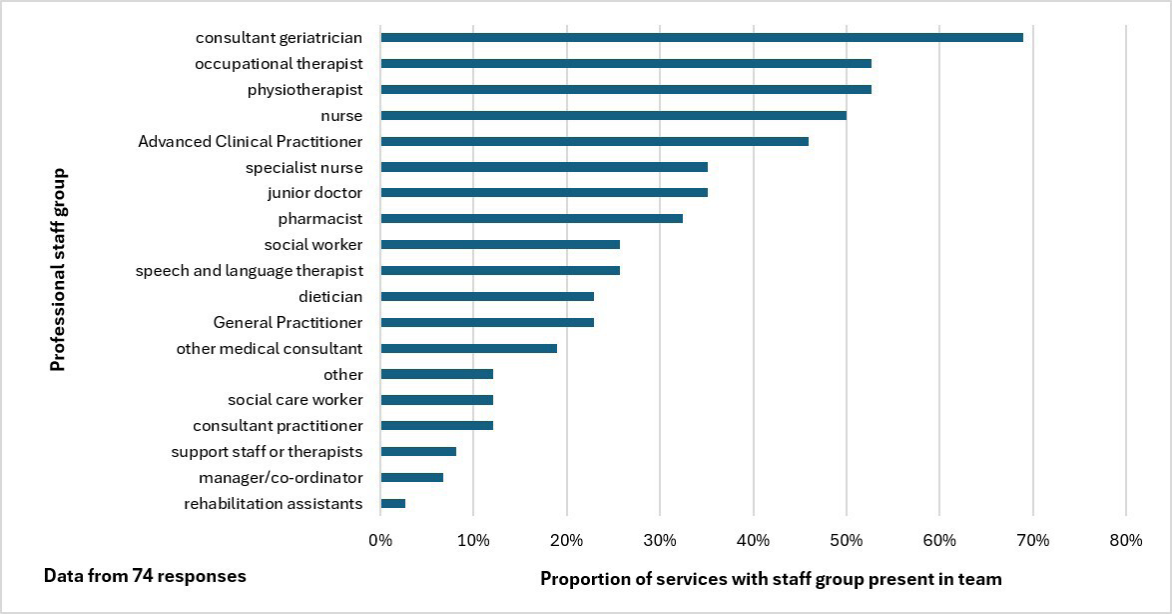
Proportion of frailty services with different professions in the delivery team.

Almost 70% of the services had a consultant geriatrician as part of the team, with more than half of the services involving a nurse, physiotherapist or occupational therapist (figure 2). Nursing or AHP staff with additional specialist training were the next most represented (35% and 46% respectively).

When services were stratified by those delivered only in secondary care (n=19) or the community (n=29), consultant geriatricians were more prevalent in secondary care frailty services than those in the community (79% vs 18%) and were often supported by resident doctors (79%). Advanced clinical practitioners and specialist nurses (ie, staff with specialist frailty training) were present in 58% and 53% of services respectively. Approximately 50% of community frailty services involved physiotherapists, nurses or occupational therapists, with advanced clinical practitioners and specialist nurses present in 45% and 31% of services. Social workers were present in 37% of secondary care services but only 21% of community services.

Around two thirds of respondents (62.5%, 30/48) stated that there were currently not sufficient human resources to deliver the service, and others reported that even though there were no vacancies, the current service staffing was lacking in core members due to insufficient budgets. Vacancies included those for both specialist and non-specialist staff, across the range of professions.

### Future frailty service priorities and workforce needs

**Figure 3:**
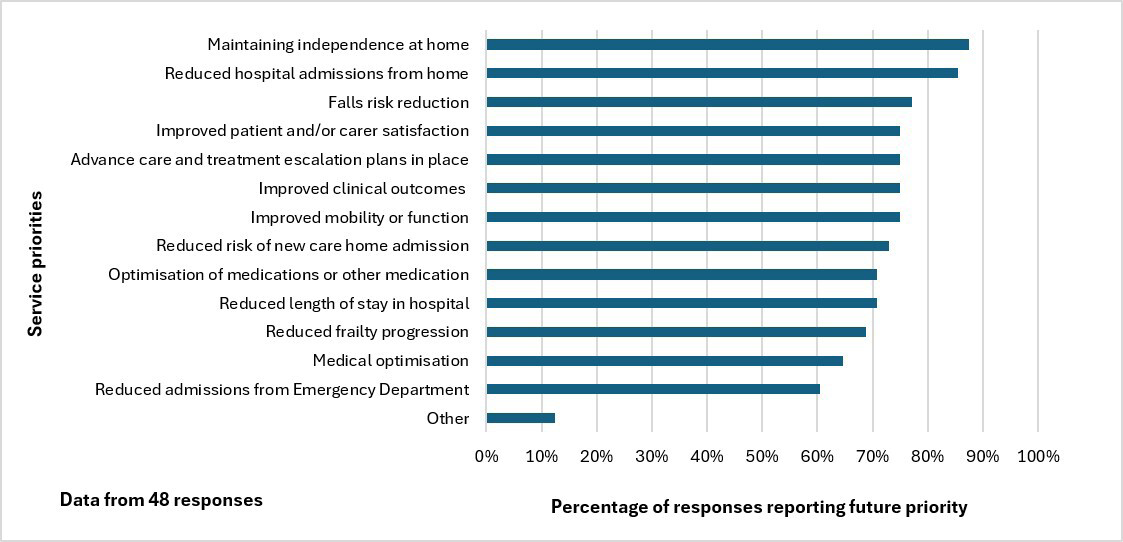
Frailty service priorities over next 5–10 years.

Maintaining independence at home and reducing hospital admissions were the most commonly reported future service priorities (figure 3). Overall, all services reported similar priorities, with additional operational-level priorities reported including widening access to the service, improving access to rapid response teams, end-of-life care, better collaboration and integration across the whole patient pathway, for example, primary and community care, improved quality of life and improved general public awareness of frailty and its sequelae.

**Figure 4:**
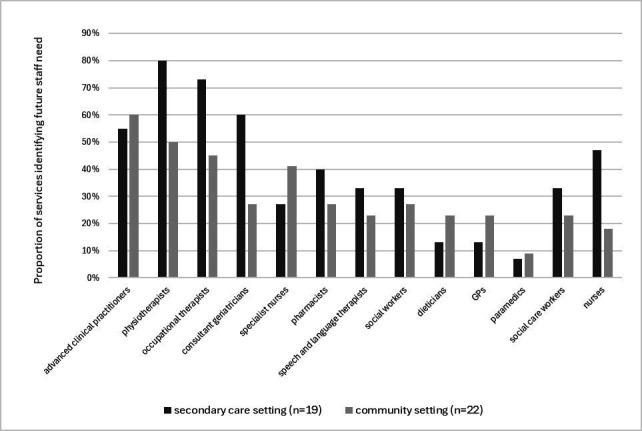
Types of staff group needed to deliver frailty service in the future according to respondents from secondary or community settings.

In response to which additional staff would be needed for the frailty service 5–10 years in the future to meet these priorities (n=47), no respondent selected ‘no change’ and all respondents selected multiple staff categories. Of the 273 options selected, almost half were non-specialist nurses or AHPs (including occupational therapists (OTs), physiotherapists, dieticians, speech and language therapists (SLTs), paramedics or pharmacists). ‘Other’ staff categories needed included discharge planning co-ordinators, rehabilitation assistants, cognitive behavioural therapy (CBT) practitioners, health and therapy assistants, exercise specialists, support staff and volunteers. When stratified by secondary-care settings and community settings where additional staff information was available, the highest needs identified secondary care were for physiotherapists, occupational therapists and consultant geriatricians, whereas respondents from community settings highlighted the need for more advanced clinical practitioners and specialist nurses, in addition to therapist input (figure 4). Both settings identified a need for more representation from social work and social care within their teams.

## Discussion

This is the first time a survey of frailty services for older people has collected information on the workforce involved in current and future service delivery. A key finding was that current services are reliant on specialist staff with knowledge and experience in care of older people, with all respondents identifying the need for several additional team members, with the types of specialists and other team members varying by delivery setting. This suggests that although current services are led by senior staff with significant clinical expertise, there is increasing recognition that broader teams are needed to deliver more rehabilitative and preventive care. Projections for future needs for geriatricians in the UK indicate significant current and future gaps,^19^ and support from an adequately trained multidisciplinary team with which tasks can be distributed may drive future service models.^20^ The survey correlates with the findings of our scoping review in that frailty specialists are often based in secondary care within multidisciplinary teams, including AHPs, with the skills needed to address different aspects of patient care.^6^ The diversity of staff types reported indicates progress from a previous survey of care for older people in the USA, which highlighted a lack of involvement from registered nurses or social workers.^25^

The expert-led model presents challenges for workforce training and funding as service locations shift more to community and primary care. Consultant geriatricians are mostly hospital-based, and so the workforce, along with the supporting organisational and employment models, will need reconfiguration to support community settings. Despite this, many geriatricians already divide their time between urgent, acute and community settings. Geriatricians with larger roles in the community or expansion of specialist nurse/ACP community roles both have implications for system-level change in the community sector and integrated care systems. It is also significant that a third of specialist staff worked only a couple of days per week in the frailty service, dividing their time between the service and other activities. This rationing of expert time, considered with the majority of services seeing people living with frailty as part of a larger service (eg, acute admissions, surgical pathways) rather than a frailty-specific service, suggests need for significant investment in community services to meet the predicted patient need and deliver against key health policy ambitions to shift from secondary to community care and prevention.^2 10^ However, a whole-systems planning approach is needed to ensure human resources are not removed from one sector to meet provision needs in other areas, without understanding the impact of these changes and the implications for governance and patient safety.

Although current frailty services span a range of activities from crisis care to ongoing management, we found that slowing frailty progression was neither a common aim nor outcome measure, while it was a high priority for future services. This supports a fundamental shift in the nature and configuration of services from reactive intervention and secondary care delivered services towards population-centred frailty prevention. Frailty services have primarily developed in the UK as a response to pressures in secondary care and the associated policy drives for frailty identification and support to reduce hospital admissions and maintain independence.^26 27^ Limited resources and focus on the most urgent consequences of frailty, exacerbated by the COVID-19 pandemic,^28^ resulted in a diversity of strategies for hospital avoidance such as Urgent Care Response teams and Hospital at Home/virtual wards dominating recent service development and resourcing priorities.^29 30^ However, our simulation model findings which identified reducing frailty incidence and slowing progression as key levers to managing demand in the long-term,^2^ and more recent policies for healthy ageing have an increased focus on public health and intersectoral working to prevent and manage frailty.^8 31^ Respondents identified the need to recruit more staff who can deliver different aspects of rehabilitative and preventive care to achieve this, for example, therapists. In future, widening the scope of services to focus on prevention of frailty progression and closer working with public health programmes relevant to frailty prevention is essential to provide more proactive care for older people and promote healthy ageing.^10 32^ New service development will inevitably lead to a range of solutions, as illustrated by the variability of operational characteristics of frailty services in emergency departments.^33^ Community care in the context of preventing and reducing frailty progression will be necessarily localised to suit the characteristics of local populations and available resources,^31^ and cross-sectoral working to embed the Health in All Policies (HiAP) approach is essential.^34^ Policy shifts from acute to community care and prevention^10^ provide an opportunity to develop appropriate services together with the patients most likely to benefit and to embed evaluation to determine what services offer, assess how and to what extent this will reduce or delay need for hospital care, and ensure equity of access and improve patient outcomes. A standardised toolkit incorporating evaluation of how pathways are operated would provide intelligence on the longitudinal impacts of frailty services and pathways and facilitate comparisons,^35^ which are essential to inform policy implementation, sustainable contracting models and multi-year investment planning.

More than half the services included end-of-life care, although few reported palliative care specialists within the team. This, together with the need to deliver more end of life care in communities, supports workforce training and development to deliver adequate specialist palliative and end-of-life care in community care settings.^36^ Advance care plans are voluntary in the UK, and were used in 44% of people dying in hospital in 2024,^37^ supporting the need for better recognition and service framework planning for end-of-life care for people with advancing frailty,^38^ along with improved care co-ordination and increased involvement of older people in developing the services they need. Similarly, the involvement of mental healthcare professionals was minimal, a finding supported by our scoping review.^6^ Although referrals to mental health teams do occur, the acknowledgement of the bi-directional role of frailty and mental health status is important to leverage involvement of such professionals within multidisciplinary frailty teams.^39 40^

Incorporation of core competencies in older people’s care and frailty into general therapist training is an important global consideration as the population continues to age, and has been recognised for some time in regards to the nursing and social care workforce working with older people.^41^ Given the multiple aims and activities reported by most respondents, staff who are able to understand and co-ordinate care across settings and enable people to receive timely care in the most efficient way are key to improving people’s care outcomes. However, it is clear that the settings in which services are placed may have different scope for integration within a wider system. For example, the variety of pathways to refer patients into the services, mostly primary, community and acute care, require clear communication as to what outcomes the service aims to provide, and involve staff working across several sectors to facilitate continuity in care.^42^ As the needs of people living with frailty change, the type of care required continues to evolve. The emerging workforce roles of care co-ordinators and continued push for integration of staff from social work/care services and support from voluntary groups become increasingly important to respond quickly to changing needs and to keep the patient and their carer/family at the heart of care objectives. Staff may also increasingly need to look outside their familiar health and care settings and care models to achieve holistic care within Neighbourhood Working frameworks and multi-agency teams to achieve joined-up care across sectors.^43^

### Limitations

Our definition of ‘frailty services’ was necessarily broad in order to capture the range of services across settings, but may have impacted on reproducibility and generalisability of the results. Future surveys may be able to more aligned to a particular type of service as policy shifts settle into standard care models. As with all surveys, the potential for selection, self-report and response biases may have impacted our recruitment and results; however, the responses represented a substantial number of different types of services and settings. It is also possible that we did not capture services delivered in locations or sectors that do not commonly use this term, for example, social care. Analysis of regional variation was not appropriate due to modest numbers in each region; however all regions highlighted similar types of service activities, so future research could focus on the variation in how these are implemented regionally. Finally, due to constraints related to anonymity, the lack of specificity in numbers of different staff members precluded definitive staffing models to be described. Although the conclusions from the survey will therefore need to be treated with caution, the survey methods were robust, and key findings aligned with our scoping review and provide information about workforce in services designed specifically for people living with frailty.

## Conclusions

Frailty services with multiple aims, often requiring input from multidisciplinary staff across several clinical and care settings and sectors, were reported by participants. As the numbers of people living with frailty and the staff needed to deliver specialised care continue to increase, workforce planning needs to ensure sufficient specialist staff supported by a multidisciplinary team with the required competencies to provide effective frailty focused care. This survey aimed to elucidate the current and planned initiatives to manage frailty, and their workforce implications. Our findings indicate a need to shift resource into community-based preventive and early-stage frailty management and progression prevention activities according to the respondent’s future service priorities. This has particular global relevance in settings where diversification of health staff may be more challenging due to resource constraints, necessitating frailty care as a priority in future workforce training for all staff.

## Supplementary material

10.1136/bmjopen-2026-116867 online supplemental file 1

## Data Availability

Data are available upon reasonable request.
