# Supplementary material for: What are the characteristics of the health and care workforce supporting people living with frailty in England now and what is needed for the future? A national survey
Source: BMJ Open. 2026 Jul 1;16(7):e116867. doi: 10.1136/bmjopen-2026-116867 (PMC13331124; doi:10.1136/bmjopen-2026-116867)
Supplement: online supplemental file 1 [file bmjopen-16-7-s001.pdf]

Introduction

Study Title: Planning for Frailty: Optimal Health and Social Care Workforce Organisation Using Demand-led Simulation Modelling (FLOWS)
Researcher: Prof. Bronagh Walsh, School of Health Sciences, University of Southampton
University contact email: F.Lambert@soton.ac.uk
Ethics: This study was approved by the Faculty Research Ethics Committee (FREC) at the University of Southampton (Ethics/ERGO Number: 71937)

What is the research about? My name is Bronagh Walsh and I am a Professor of Health Care for Older People at the University of Southampton in the United Kingdom. I am inviting you to participate in a study regarding improving understanding of health and social care service demand related to frailty, and how the size and competencies of the workforce need to be developed to meet demand. The over-arching aim is to understand the present, and expected, size and composition of health and social care workforce requirements associated with optimal care provision for

the frail older population. The study will bring together evidence from a review of the literature, consultation with stakeholders, analysis of routine health and social care data and simulation modelling. As part of the consultation with professionals involved in providing services to older people with frailty, we are inviting you to participate in this survey. The survey will help us address the following research questions: What is known about the workforce required to deliver health and social care services? What services are currently provided or being developed for prevention and management of frailty and what are the associated requirements in terms of workforce numbers, skills and competencies?

What will happen to me if I take part? This study involves completing an anonymous questionnaire which should take approximately 15 minutes of your time. You will be able to start completing the survey, and your answers will be saved so that you can return to it later, rather than have to complete the whole survey at one sitting. If you are happy to complete this survey, you will need to answer the question below, to confirm your eligibility and then tick the box below to show your consent. As this survey is anonymous, the research team will not be able to know whether you have participated, or what answers you provided , and your .data cannot be withdrawn after submission.

Why have I been asked to participate? You have been

sent this invitation to participate because you are a health care, public health or social care professional who is involved in planning or delivering care for older people living with or at risk of frailty. We are aiming to recruit around 200 participants from England and Wales for this study.

What information will be collected? The questions in this survey ask for information in relation to your professional group, the health or social care sector you work in, the type of service you provide for people living with or at risk of frailty and your experience and qualifications. You will also be asked to provide information on the service size/caseload, other professions involved, numbers and types of staff and vacant posts. We will not ask for personally identifying information. You do not have to answer all the questions if you do not wish to do so.

What are the possible benefits of taking part? If you decide to take part in this study, you will not receive any direct benefits; however, your participation will contribute to knowledge in this area of research, which will inform workforce planning for older people living with frailty.

Are there any risks involved? It is expected that taking part in this study will not cause you any psychological discomfort and/or distress, however, should you feel uncomfortable you can leave the survey at any time or

leave a question uncompleted.

What will happen to the information collected? All information collected for this study will be stored securely on a password protected computer and backed up on a secure server. In addition, all data will be pooled and only compiled into data summaries or summary reports. Only the research team will have access to raw data from this study. The University of Southampton conducts research to the highest standards of ethics and research integrity. In accordance with our Research Data Management Policy, data will be held for 10 years after the study has finished when it will be securely destroyed. What happens if there is a problem? If you are unhappy about any aspect of this study and would like to make a formal complaint, you can contact the Head of Research Integrity and Governance, University of Southampton, on the following contact details: Email: rgoinfo@soton.ac.uk, phone: + 44 2380 595058. Please quote the Ethics/ERGO number above. Please note that by making a complaint you might be no longer anonymous. More information on your rights as a study participant is available via this link: https://www.southampton.ac.uk/about/governance/partic information.page

Thank you for reading this information sheet and considering taking part in this research.

Are you involved in the provision of a service for older people that are living with frailty?

- ○ No
- ○ Yes

Thank you for reading this information and considering taking part in this research.

○ Please tick (check) this box to indicate that you have read and understood this information , are aged 18 or over and agree to take part in this survey.

Thank you for considering taking part in this research, but you do not meet our criteria for participation. You do not need to do anything further.

About your role in the frailty service

About your role in the frailty service

Which of the following best describes your role within the service for older people living with frailty?

- Individual service provider
- Service leader
- Team leader
- Team member
- Service manager or director
- Other (please specify)

Which of the following best describes your professional group/disciplinary background?

- Nurse
- Specialist nurse
- Advanced clinical practitioner
- Consultant practitioner
- GP
- Junior doctor
- Consultant geriatrician
- Other medical consultant
- Physiotherapist
- Occupational therapist
- Speech and language therapist
- Dietician

- ○ Paramedic
- ○ Pharmacist
- ○ Social worker
- ○ Social care worker
- ○ [ ] Other (please specify)

How many years' experience do you have of working with older people living with frailty?

- ○ Up to 3 years
- ○ 4-10 years
- ○ More than 10 years

How many hours a week do you work in this service? (If in an embedded or liaison service, please give the number of hours spent in the frailty service only)

[ ]

How long has your service for older people living with frailty been running?

- ○ Less than 1 year
- ○ 1-2 years

- ○ 3-5 years
- ○ More than 5 years

In which settings is your service located? (Tick all that apply).

- ☐ Primary care
- ☐ Community care
- ☐ Mental health
- ☐ Acute hospital (inpatient)
- ☐ Emergency Department
- ☐ Acute hospital (outpatient)
- ☐ Community hospital (inpatient)
- ☐ Community hospital (outpatient)
- ☐ Ambulance service
- ☐ Prison
- ☐ Integrated care system
- ☐ Residential or nursing home
- ☐ Learning disability
- ☐ Charity or voluntary
- ☐ [ ] Other (please specify)

Which NHS England region is your service located in?

- ○ North West
- ○ North & East Yorkshire

- ○ Midlands
- ○ East of England
- ○ South West
- ○ South East
- ○ London

What specialist qualifications do you have in care of older people/older people's medicine/frailty? Please specify:

[ ]

What is your highest educational qualification?

- ○ Undergraduate diploma
- ○ Undergraduate degree
- ○ Postgraduate degree
- ○ MD or PhD
- ○ [ ] Other (please specify)

About your frailty service

About your frailty service

Which types of patients is your service for?

- ○ Older people at risk of developing frailty
- ○ Older people generally, some of whom could be living with frailty
- ○ Only people identified as living with frailty
- ○ People identified as frail within another service, e.g. orthopaedics, urology, oncology, ED (please specify) [ ]

What are the main activities of your service? (Tick all that apply).

- ☐ Frailty prevention
- ☐ On-going frailty management
- ☐ Reducing risks associated with frailty (e.g. falls, hospital admission, poor care outcomes)
- ☐ End of life care
- ☐ Frailty identification and onward referral
- ☐ Rehabilitation or re-ablement (hospital based)
- ☐ Rehabilitation or re-ablement (home based)
- ☐ Intermediate care
- ☐ Urgent or crisis care
- ☐ [ ] Other (please specify)

What tools are used to identify frail older people referred to/ offered your service?

- [ ] Rockwood clinical frailty scale (CFS)
- [ ] Fried criteria
- [ ] Gait Speed Test
- [ ] PRISMA-7
- [ ] Timed Up and Go test
- [ ] Electronic Frailty Index
- [ ] Other (please specify) ____________

If patients are referred to your service, where do your service referrals come from? (Tick all that apply).

- [ ] GP practice
- [ ] Community services, including nursing
- [ ] Social care
- [ ] Voluntary services
- [ ] Secondary care
- [ ] Urgent care
- [ ] Self-referral
- [ ] Other (please specify) ____________

What is the average caseload (patients seen per week) for a team / staff member in your service?

What are your key measures of success? (Tick all that apply).

- ☐ Reduced frailty progression
- ☐ Improved mobility or function
- ☐ Medical optimisation
- ☐ Maintaining independence at home
- ☐ Falls risk reduction
- ☐ Reduced hospital admissions from home
- ☐ Reduced risk of new care home admission
- ☐ Reduced admissions from ED
- ☐ Reduced length of stay in hospital
- ☐ Improved clinical outcomes (e.g. death in hospital, falls, re-admission)
- ☐ Advance care and treatment escalation plans in place
- ☐ Optimisation of medications or other treatments
- ☐ Improved patient and / or carer satisfaction
- ☐ Other (please specify)

What should be frailty service priorities over the next 5-10 years? (Tick all that apply).

- ☐ Reduced frailty progression
- ☐ Improved mobility or function
- ☐ Medical optimisation
- ☐ Maintaining independence at home
- ☐ Falls risk reduction
- ☐ Reduced hospital admissions from home
- ☐ Reduced risk of new care home admission
- ☐ Reduced admissions from ED
- ☐ Reduced length of stay in hospital
- ☐ Improved clinical outcomes (e.g. death in hospital, falls, re-admission)
- ☐ Advance care and treatment escalation plans in place
- ☐ Optimisation of medications or other treatments
- ☐ Improved patient and / or carer satisfaction
- ☐ Other (please specify) ________

About the staff needed to run your frailty service

About the staff needed to run your frailty service

Who else is involved in delivering your service? (Tick all that apply).

- ☐ Nurse
- ☐ Specialist nurse
- ☐ Advanced clinical practitioner
- ☐ Consultant practitioner
- ☐ GP
- ☐ Junior doctor
- ☐ Consultant geriatrician
- ☐ Other medical consultant
- ☐ Physiotherapist
- ☐ Occupational therapist
- ☐ Speech and language therapist
- ☐ Dietician
- ☐ Paramedic
- ☐ Pharmacist
- ☐ Social worker
- ☐ Social care worker
- ☐ __________ Other (please specify)

Do you have sufficient staff resources to deliver your service?

- ○ Yes
- ○ No

If no, specify number of WTE vacancies and staff type

[ ]

What additional staff resources are needed to deliver your service 5-10 years into the future? (Tick all that apply)

- [ ] No change
- [ ] More nurses
- [ ] More specialist nurses
- [ ] More advanced clinical practitioners
- [ ] More consultant practitioners
- [ ] More GPs
- [ ] More junior doctors
- [ ] More consultant geriatricians
- [ ] More other medical consultants
- [ ] More physiotherapists
- [ ] More occupational therapists
- [ ] More speech and language therapists
- [ ] More dieticians
- [ ] More paramedics
- [ ] More pharmacists
- [ ] More social workers
- [ ] More social care workers

☐ Other (please specify) ______

Powered by Qualtrics
