# Supplementary material for: What are the characteristics of the health and care workforce supporting people living with frailty in England now and what is needed for the future? A national survey
Source: BMJ Open. 2026 Jul 1;16(7):e116867. doi: 10.1136/bmjopen-2026-116867 (PMC13331124; doi:10.1136/bmjopen-2026-116867)
Supplement: Supplementary file 2 [file bmjopen-2026-116867.reviewer_comments.pdf]

PEER REVIEW HISTORY

BMJ Open publishes all reviews undertaken for accepted manuscripts. Reviewers are asked to complete a checklist review form and are provided with free text boxes to elaborate on their assessment. These free text comments are reproduced below.

ARTICLE DETAILS

Title (Provisional)

What are the characteristics of the health and care workforce supporting people living with frailty in England now and what is needed for the future? A national survey

Authors

Fogg, Carole; England, Tracey; Vernon, Martin; Ball, Jane; Fenge, Lee-Ann; Griffiths, Peter; Lambert, Francesca; Barkham, Abigail; Brailsford, Sally C.; Windle, Vivienne; Walsh, Bronagh

---

VERSION 1 - REVIEW

**Reviewer**	**1**
**Name**	**Susanto, Tantut**
**Affiliation**	**Universitas Jember, Community, Family, Geriatric Nursing**
**Date**	**17-Feb-2026**
**COI**	**None**

Dear Authors,

The manuscript, "What is the current and future health and care workforce supporting people living with frailty in England? A survey," addresses an important issue in the future of frailty prevention. However, several points require discussion.

1. Background. The issue of frailty is a very common issue related to chronic conditions or issues related to aging. Therefore, it is necessary to describe the urgency of this issue in relation to the current state of frailty and its future importance. It also describes how prevention is implemented in healthcare services, what is currently available, and what frailty services and care might look like in the future, if well prepared. Therefore, it is also necessary to support this with empirical studies related to this issue, including what the gold standard in frailty care is like, and what are its weaknesses?

2. Method. As a cross-sectional study, it is best to clarify the study according to the STROBE guidelines. For example, the estimated sample size for this survey, the response rate, and the inclusion and exclusion criteria need to be clarified. For cross-sectional studies, it's best to explain when the study was conducted and, as a national survey, how each survey unit

was involved in data collection. The variables to be analyzed should be clarified based on the research problem and the results of previous empirical studies addressing the research question "What is the current and future health and care workforce supporting people living with frailty in England? A survey."

3. Results. Generally, the data reflects the research problem. However, the data visualization should be more informative and communicative (not necessarily in tables, so the results can be more easily read as national survey results).

4. Discussion. It's best to present the research findings and their current findings and their general implications for the development of frailty care service needs.

5. Conclusion. A restatement of the research objectives is needed.

Thank you.

Sincerely yours,

Reviewer

**Reviewer**	**2**
**Name**	**Zeidan, Rola S**
**Affiliation**	**University of Florida, Physiology and Aging**
**Date**	**22-Mar-2026**
**COI**	**None**

The manuscript addresses an important and timely gap in understanding workforce needs for frailty services, with clear relevance to health system planning in aging populations. However, the manuscript could benefit from addressing the following comments:

1- Sampling approach is non-systematic - the representativeness of responding services relative to all frailty services in England should be clarified. Also, although not major but the extended survey period may introduce temporal variability in responses, esp with workload differences -- make sure you acknowledge this.

2- Definition and categorization of “frailty services” are not clearly operationalized --greater clarity would improve interpretability and reproducibility, especially that many services are mixed. Need to clarify and clearly acknowledge since this might affect interpretability of results.

3- Limited attention is given to regional variation in service provision and workforce needs, which could provide valuable insight for policy and planning. Suggest analyzing or interpreting regional variation, not just reporting it.

4- Need to clarify how duplicate or overlapping service responses were handled. Are there potentially more than 1 response per service? If yes, how did you deal with that?

5- Tables reported are very helpful but suggest adding clearer synthesis of the data – Also, analysis is mostly descriptive. Additionally, tables appear to be missing denominator of total services. Also maybe consider adding a single integrated summary table (services + workforce + gaps).

6- The finding that services focus more on risk reduction than prevention is important - but the manuscript could better explore potential underlying reasons).

7- The role of non-specialist staff is highlighted but not deeply interpreted -- maybe this could be framed within models of multidisciplinary care and task-shifting.

8- Conclusions would benefit from more cautious wording, since findings are based on self-reported perceptions rather than objective workforce metrics.

9- The manuscript would be strengthened by a more structured framework or model summarizing optimal workforce composition for frailty care.

10- For limitations, need to emphasize the following: Potential selection and response bias due to survey distribution method - Reliance on self-reported data rather than objective workforce measures - Lack of granularity in staffing data due to anonymity constraints.

11- Minor comments: Need to refine language in the conclusion to be more neutral and evidence-aligned. Need to clarify terminology for an international audience. Add a table summarizing workforce composition and service characteristics to improve clarity.

VERSION 1 - AUTHOR RESPONSE

Reviewer: 1
Dr. Tantut Susanto, Universitas Jember
Comments to the Author:
Dear Authors,

The manuscript, "What is the current and future health and care workforce supporting people living with frailty in England? A survey," addresses an important issue in the future of frailty prevention. However, several points require discussion.

1. Background. The issue of frailty is a very common issue related to chronic conditions or issues related to aging. Therefore, it is necessary to describe the urgency of this issue in relation to the current state of frailty and its future importance. It also describes how prevention is implemented in healthcare services, what is currently available, and what frailty services and care might look like in the future, if well prepared. Therefore, it is also necessary to support this with empirical studies related to this issue, including what the gold standard in frailty care is like, and what are its weaknesses?

Response: Thank you. We have edited the first paragraph of the Introduction to reflect the urgency and the gap between need and demand. We have also highlighted the lack

of a ‘gold standard’ definition of care, which further complicates the issue of understanding the required workforce.

2. Method. As a cross-sectional study, it is best to clarify the study according to the STROBE guidelines. For example, the estimated sample size for this survey, the response rate, and the inclusion and exclusion criteria need to be clarified. For cross-sectional studies, it's best to explain when the study was conducted and, as a national survey, how each survey unit was involved in data collection. The variables to be analyzed should be clarified based on the research problem and the results of previous empirical studies addressing the research question "What is the current and future health and care workforce supporting people living with frailty in England? A survey."

Response: Thank you – as guided by the Editor’s recommendations, we have now completed a CROSS checklist which is designed for surveys. As this survey was sent out via networks of professionals involved in frailty care, there is no denominator with which to calculate a response rate. Although these networks exist at a national level, hence the ‘national survey’ in the title, we did not aim to collect complete data across the various care settings, but aimed to elucidate the current and planned initiatives to manage frailty, and their workforce implications, as outlined in the objectives at the end of the introduction. The section on ‘Distribution’ in the Methods should hopefully clarify this. We have found no evidence of previous surveys examining the workforce in this area.

3. Results. Generally, the data reflects the research problem. However, the data visualization should be more informative and communicative (not necessarily in tables, so the results can be more easily read as national survey results).

Response: thank you – we have used a combination of Tables and Figures according to the type of data presented – the Figures were in a separate upload at the end of the manuscript rather than in the text, as per the journal guidance. Hopefully these will be accessible to you in the revised version.

4. Discussion. It's best to present the research findings and their current findings and their general implications for the development of frailty care service needs.
Response: Thank you – we have reviewed the discussion and believe that it meets the description above.

5. Conclusion. A restatement of the research objectives is needed.

Response: thank you – a sentence has been added to the conclusions which summarises the survey aims, prior to the summary of the findings.

Thank you.

Sincerely yours,
Reviewer

Reviewer: 2
Dr. Rola S Zeidan, University of Florida
Comments to the Author:
The manuscript addresses an important and timely gap in understanding workforce needs for frailty services, with clear relevance to health system planning in aging populations. However, the manuscript could benefit from addressing the following comments:
1- Sampling approach is non-systematic - the representativeness of responding services relative to all frailty services in England should be clarified. Also, although not major but the extended survey period may introduce temporal variability in responses, esp with workload differences -- make sure you acknowledge this.

Response: thank you for raising this. Given the very localised development of frailty services and their positioning across different aspects of the health system, there is no available register of all existing services and it was not therefore possible to use sampling strategy or capture a representative sample. We therefore utilised professional networks focused on capturing the range of services and settings providing or developing these services across the country. This has been clarified in the methods. We feel this aim has been met by the responses given. As outlined at the end of the introduction section, this survey was a component of a larger project gathering data through different methods to contribute to a simulation modelling study, so the information from this survey is appropriate to meet the needs of the project.

We appreciate the point regarding temporal variability, but we consider that it has limited relevance for this survey where we are looking at long-term staff needs and team composition that would not be likely to change within the extended survey period.

2- Definition and categorization of “frailty services” are not clearly operationalized -- greater clarity would improve interpretability and reproducibility, especially that many services are mixed. Need to clarify and clearly acknowledge since this might affect interpretability of results.

Response: Thank you – this is partly to the difficulty of a standard package of care for frailty services and a standard definition which applies across settings, and partly due to our aim of capturing new and developing services as well as established ones. It was

important to allow respondents to identify their contribution and not to rely on existing models of care. We have edited the ‘participants’ section in the methods to reflect this, and also at the beginning of the ‘limitations’ section. We gathered data in the survey to capture the main activities of the services, including core aspects of frailty care i.e. reduction of risks associated with frailty, management, end-of-life care, urgent or crisis care, identification and referral, prevention and intermediate care (as presented in Figure 1). As expected, most services covered several of these key areas, highlighting the difficulty of establishing a standard definition.

3- Limited attention is given to regional variation in service provision and workforce needs, which could provide valuable insight for policy and planning. Suggest analyzing or interpreting regional variation, not just reporting it.

Response: thank you for this important point. It is very true that the localised service developments may demonstrate significant regional variation. However, although our responses from some regions were more than others, we don’t feel the sample is sufficient to analyse by region and draw reputable conclusions. We have included this in the limitations and as an area for future investigation.

4- Need to clarify how duplicate or overlapping service responses were handled. Are there potentially more than 1 response per service? If yes, how did you deal with that?

Response: As the survey link was sent to unknown participants via organisations and the responses were anonymous, we were unable to prevent multiple participation of participants, although we considered this unlikely. However, we assessed responses for potential duplicates by examining the participant characteristics (e.g. region, profession, role) and did not find anything to suggest that the survey had been completed multiple times by the same respondent. It is also possible (although unlikely) that we gained responses from more than one person working within the same service, in which case this would have added to the depth of data on team composition and priorities, which could differ between different team members or professions, so could be considered useful data.

5- Tables reported are very helpful but suggest adding clearer synthesis of the data – Also, analysis is mostly descriptive. Additionally, tables appear to be missing denominator of total services. Also maybe consider adding a single integrated summary table (services + workforce + gaps).

Response: Thank you for this suggestion. We had also included 4 figures which highlight some of the key points and are unsure if these were provided at peer review stage. We have inserted the overall total of responses for Table 3 where it was missing,

the denominator for the other tables and figures has been given as number of responses where appropriate. Please refer to the revised manuscript for these. A single integrated summary table would be difficult to achieve, given the multiple response nature of several of the key questions, necessitating quite a complex multidimensional structure. We hope that the included Figures add to the information in the tables to illustrate the key points.

6- The finding that services focus more on risk reduction than prevention is important - but the manuscript could better explore potential underlying reasons).

Response: Thank you. We have edited the third paragraph of the discussion to highlight the reasons for the original focus and shift in policy and hope that this is now clearer (although we are limited in additional detail by the word count).

7- The role of non-specialist staff is highlighted but not deeply interpreted -- maybe this could be framed within models of multidisciplinary care and task-shifting.

Response: Thank you – we have edited the first paragraph of the discussion to reflect the gaps within the workforce for specialist staff and the opportunities for the multidisciplinary team in future service models.

8- Conclusions would benefit from more cautious wording, since findings are based on self-reported perceptions rather than objective workforce metrics.

Response: Thank you – we have included an indication in the conclusions that the findings are from participant reports and altered the wording to be more cautious.

9- The manuscript would be strengthened by a more structured framework or model summarizing optimal workforce composition for frailty care.

Response: Thank you for this suggestion. We feel that this is outside of the scope of this manuscript for several reasons. The intention of the survey was to gather information on the activities of frailty services, newly developed or planned services, and the associated professionals providing the service and identify workforce gaps and future directions to inform stakeholder discussions to build a simulation model for a frailty-specific workforce. However, as also alluded to above in the response to Question 2, although guidance relating to service design and workforce exists, there is no clear standardisation of services and no optimal workforce composition has been tested or agreed. Our hope is that this manuscript presents data to inform that conversation and to set the findings within the context of current policy shifts, but we cannot utilise our data to define optimal models.

10- For limitations, need to emphasize the following: Potential selection and response bias due to survey distribution method - Reliance on self-reported data rather than objective workforce measures - Lack of granularity in staffing data due to anonymity constraints.

Response: Thank you – we have edited the limitations as suggested.

11- Minor comments: Need to refine language in the conclusion to be more neutral and evidence-aligned. Need to clarify terminology for an international audience. Add a table summarizing workforce composition and service characteristics to improve clarity.
Response: Thank you – we have adjusted the conclusion as outlined in the above responses and clarified terminology such as GP=primary care doctor within the list of abbreviations. Please see above responses regarding tables/figures.

VERSION 2 - REVIEW

**Reviewer**	**1**
**Name**	**Susanto, Tantut**
**Affiliation**	**Universitas Jember, Community, Family, Geriatric Nursing**
**Date**	**31-May-2026**
**COI**	

Dear Authors,

This revised edition of the manuscript, "What are the characteristics of the health and care workforce supporting people living with frailty in England now and what is needed for the future? A national survey," generally incorporates input and improvements from the reviewers' comments. This manuscript is improved and has been improved in its contribution to client care with frailty. Some additional data and study limitations have also been well described. Thank you.

Best regards,

Reviewer

**Reviewer**	**2**
**Name**	**Zeidan, Rola S**
**Affiliation**	**University of Florida, Physiology and Aging**
**Date**	**06-Jun-2026**

COI

Authors have responded adequately to almost all comments. Minor changes requested -

manuscript still requires a clearer operational definition of what qualified as a “frailty service,” including to briefly mention whether inclusion was based on self-identification, what was referral pathway, service name if possible... The authors should also clarify how mixed services were handled analytically, including whether services were assigned to one primary category, allowed to belong to multiple categories, or analyzed according to activity domains. Also although authors replied to comment 4 - it has to be clearly acknowledged and mentioned in manuscript methods.

VERSION 2 - AUTHOR RESPONSE

Reviewer: 1
Dr. Tantut Susanto, Universitas Jember
Comments to the Author:
Dear Authors,

This revised edition of the manuscript, "What are the characteristics of the health and care workforce supporting people living with frailty in England now and what is needed for the future? A national survey," generally incorporates input and improvements from the reviewers' comments. This manuscript is improved and has been improved in its contribution to client care with frailty. Some additional data and study limitations have also been well described. Thank you.

Best regards,
Reviewer

Response: Many thanks for your time reviewing our paper and your useful comments.

Reviewer: 2
Dr. Rola S Zeidan, University of Florida
Comments to the Author:
Authors have responded adequately to almost all comments. Minor changes requested -
1. manuscript still requires a clearer operational definition of what qualified as a “frailty service,” including to briefly mention whether inclusion was based on self-identification, what was referral pathway, service name if possible...

Response: Thank you. The “Participants” section of the manuscript has been further updated to include the specific eligibility question, i.e. “Are you involved in the provision of a service for older people that are living with frailty?” The text has also been edited to further explain why this was necessary – i.e. the different development of services for people with frailty according to local needs and other features of local health and care systems. As explained

previously, due to the evolving nature of frailty in the population and local solutions to manage needs, there is no standardisation of these services, and so it was not possible to define a frailty service according to specific features e.g. sector of the health system or referral pathway. Therefore we used this question to be as inclusive as possible.

2. The authors should also clarify how mixed services were handled analytically, including whether services were assigned to one primary category, allowed to belong to multiple categories, or analyzed according to activity domains.

Response: Although some services crossed healthcare settings, the majority of the survey answers were focussed on aspects of the service delivery which did not require a formal categorisation of services. Indeed, due to the diversity of service models and the variety of activities present within a single service, it would be very difficult to devise specific categories. There is only one area where services were stratified according to care setting (secondary care or community care only) at the end of the ‘current workforce configurations and patient caseloads’ section of the results. This has now been clarified at the end of the Methods: Analysis section.

3. Also although authors replied to comment 4 - it has to be clearly acknowledged and mentioned in manuscript methods.

Response: Thank you – we have used the previous response to question 4 to further clarify this in the Methods: Analysis section.
